# Pathways of introduction of the invasive aquatic plant *Cabomba caroliniana*

**DOI:** 10.1002/ece3.530

**Published:** 2013-04-15

**Authors:** Andrée McCracken, Jillian D Bainard, Michelle C Miller, Brian C Husband

**Affiliations:** Department of Integrative Biology, University of GuelphGuelph, Ontario, Canada, N1G 2W1

**Keywords:** Fanwort, genome size, haplotype network, invasive species, molecular variation, polyploidy

## Abstract

The pathway and frequency of species' introductions can affect the extent, impact, and management of biological invasions. Here, we examine the pathway of introduction of the aquatic plant *Cabomba caroliniana* (fanwort) into Canada and the northern United States using plastid DNA sequence (intergenic spacers *atpF*-*atpH*, *trnH*-*psbA,* and *trnL*-*trnF*) and DNA content analyses. We test the hypothesis that the spread of fanwort is a result of commercial trade by comparing a Canadian population (Kasshabog Lake, ON) to native populations from southern U.S., introduced populations in northern U.S., and plants from commercial retailers. Thirteen plastid haplotypes were identified throughout North America, including one dominant haplotype, which was present in all *C. caroliniana* populations. Several rare haplotypes were used to infer shared colonization history. In particular, the Canadian population shared two rare alleles with a population from Massachusetts, suggesting range expansion of *C. caroliniana* from the northern U.S. However, the possibility of a commercial introduction cannot be excluded, as common alleles were shared between the Canadian population and both commercial and southern U.S. sources. Variation in *C. caroliniana* genome size was bimodal and populations were classified into “high” and “low” categories. The Canadian population had DNA contents similar to several northern U.S. populations (low DNA content). This may provide additional support for range expansion from these introduced populations rather than from commercial sources or populations in the southern U.S., which had high DNA content.

## Introduction

Invasive species are nonnative organisms that are capable of colonizing, establishing, and spreading significantly within natural communities (Les and Mehrhoff 1999). To better understand the mechanisms of invasiveness, researchers have focused on the attributes of invasive species in their introduced range (Baker 1965; Devin and Beisel 2007; Wilson et al. 2007), impacts on the native community (Callaway and Maron 2006; Kotta et al. 2006; Briggs 2007; Hogsden et al. 2007), and mechanisms of control and management (Hanlon et al. 2000; Li and Ye 2006; Schooler et al. 2006). More recently, with the advent of molecular markers, researchers are reconstructing the history of invasion and focusing on changes in genetic structure and ecology during invasion (e.g., Durka et al. 2005; Andreakis et al. 2007; Besnard et al. 2007; O'Doherty and Sherwood 2007; Doorduin et al. 2010; Zhang et al. 2010). Specifically, observations of genetic variation can provide insight into the pathways of introduction of invasive species (e.g., Besnard et al. 2007; Marrs et al. 2008; Okada et al. 2009; Azuma et al. 2011; Silva-Rocha et al. 2012).

The pathway of introduction of a species from a source population into a given area refers to the number of independent introductions and the mechanism(s) of introduction. These attributes determine the range of genotypes available to colonize an area. The introduced genotypes can affect the diversity of phenotypes present in the introduced range (Vellend et al. 2009), which can in turn influence the ecological tolerances of an introduced population, its likelihood and rate of subsequent expansion, and its effects on native species diversity (e.g., Culley and Hardiman 2007; Lavergne and Molofsky 2007; Facon et al. 2008). The pathway of introduction can contribute to the invasion success of a species (Wilson et al. 2009; Pyšek et al. 2011; Rago et al. 2012), and knowledge of the number and location of source populations may provide information about the ability of an invading species to spread and help to predict the severity of its impacts (Estoup and Guillemaud 2010). These factors are all important in developing appropriate management strategies to prevent invasions. In aquatic systems, the use of molecular tools has been particularly useful in determining the pathways of introduction of invasive species (e.g., Eckert et al. 2003; Azuma et al. 2011; Ghabooli et al. 2011; Lejeusne et al. 2011; Thum et al. 2011; Blanchet 2012).

Variation in DNA content could also provide a way to trace pathways of introduction. If intraspecific variation in DNA content exists within a species, then that variation may be a useful tool for inferring evolutionary history, especially in cases where there are known differences in ploidy (Kron et al. 2007). Polyploid formation is relatively rare and heritable (Ramsey and Schemske #b^503^) allowing inferences to be made about source populations. For example, ploidy variation was used to help identify the source of a recently colonized population of *Cardamine* in Belgium (Bleeker et al. 2008). Intraspecific variation in DNA content not due to ploidy may also offer insights into colonization history. Although it has often been attributed to methodological or environmental artifacts (see Greilhuber 2005), support for heritable intraspecific variation does exist (e.g., Šmarda 2006; Šmarda and Bureš 2006; Loureiro et al. 2007), and it can be correlated with geographic location (Šmarda 2006). Intraspecific variation, therefore, may provide additional insight into the pathways of introduction.

**Figure 1:**
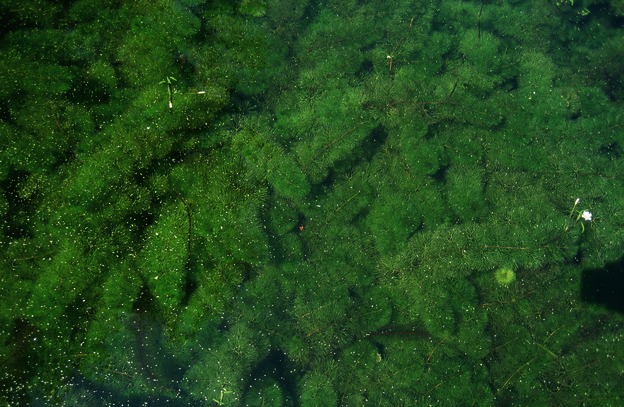
*Cabomba caroliniana* A. Gray (fanwort) forming a characteristic mat of vegetation in a lake. (Photo credit: Andrée McCracken)

**Figure 2:**
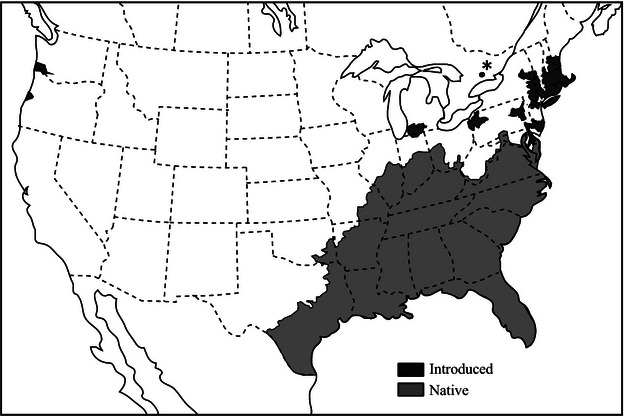
Distribution of established *Cabomba caroliniana* populations in the United States and Canada. Shaded regions correspond to observed distribution. Adapted from Cao (2012). The Canadian population in Kasshabog Lake, Ontario, is marked with an asterisk.

*Cabomba caroliniana* A. Gray (fanwort) is a subtropical freshwater submerged aquatic plant (Fig. 1) that is spreading worldwide. It is native to South America and is considered native or naturalized in the southeastern United States (Mackey and Swarbrick 1997; Wilson et al. 2007). Introduced populations of *C. caroliniana* exist worldwide, in Australia, China, Japan, New Guinea, Malaysia, and several European countries (Wilson et al. 2007; Brunel et al. 2010). In the United States, introduced populations of *C. caroliniana* have become established in several northeastern states and in Washington and Oregon (Fig. 2, Cao 2012) and it has also been documented in California (Hrusa et al. 2002). Although it is widespread in the United States, *C. caroliniana* currently has a limited distribution in Canada. The only known established population was discovered in 1991 in Kasshabog Lake near Peterborough, Ontario (Hogsden et al. 2007). Since 1991, *C. caroliniana* has spread down the North River to nearby South Lake and Big Bass Bay (Wilson et al. 2007). In its introduced range, *C. caroliniana* forms dense monotypic stands that can have a significant effect on macrophyte composition and human recreational activities (Les and Mehrhoff 1999; Lyon and Eastman 2006; Hogsden et al. 2007; Wilson et al. 2007). Chromosome counts of *C. caroliniana* have revealed polyploid (3×, 6×, 8×) and aneuploid variation between individuals (Ørgaard 1991).

Little is known about the pathways of introduction of *C. caroliniana*, but it is largely suspected to be human mediated. The initial introduction of *C. caroliniana* to the northern United States is suggested to be an escape from cultivation (Les and Mehrhoff 1999). As *C. caroliniana* is still commonly sold in garden centers and aquarium stores worldwide, introduction from commercial sources (via intentional cultivation or unintentional disposal of aquarium contents) remains a plausible source of newly discovered populations (e.g., Les and Mehrhoff 1999; Kerr et al. 2000). This claim has led some states in the United States, such as California, Maine, Washington, and Connecticut, to ban the sale of this species (United States Department of Agriculture Natural Resources Conservation Service 2007). However, this hypothesis has never been rigorously tested. While it seems likely that commercial trade could facilitate global transport, the mechanisms of spread within continents are less clear. In North America, it is equally possible that the spread of *C. caroliniana* into northern United States and Canada is a range expansion from established populations in the United States via dispersal by wildlife or recreational boaters (Les and Mehrhoff 1999; Hogsden et al. 2007; Wilson et al. 2007). The goal of this study was to identify the primary pathway of introduction of *C. caroliniana* in North America. Specifically, we tested three main hypotheses regarding the source of the Canadian population: (1) accidental or intentional introduction from a commercial source, (2) introduction from populations in the northeastern United States, or (3) introduction from both sources. Two types of genetic marker were used to study the colonization history of *C. caroliniana*: chloroplast sequence data and genome size.

## Methods

### Sample collection

*Cabomba caroliniana* was sampled in 2008 from four major sources: (1) retail commercial supply stores and garden centers in Canada and the United States; (2) introduced range in the northeastern United States; (3) native range in the southern United States; and (4) introduced range in Canada. One to three live ornamental plants were purchased from each of 13 commercial outlets in Canada and five in the northern United States, and dried plant tissue from a single aquarium store in Florida was received. Eighteen wild populations were sampled, and where possible, a minimum of 20 samples were collected from each population; however, this number varied depending on *C. caroliniana* abundance (Table 1). Since the recent expansion into Canada was the main focus of the study, Kasshabog Lake was sampled more intensively, with 10 samples collected from each of 10 bays throughout the lake. As *C. caroliniana* can reproduce clonally, samples were collected a minimum of 10 m apart in an attempt to maximize the genetic diversity sampled within a population. Upon collection, tissue was immediately dried and preserved in silica gel for genetic analysis. In addition, between one and three live plants were collected from Kasshabog Lake, the sites in northeastern United States, and all commercial sources (except Florida). These live plants were grown in a common greenhouse environment to test the effect of environment on genome size estimates (see below).

**Table 1:** Locations of all *Cabomba caroliniana* populations sampled, along with the identifying code used throughout the article. The total number of samples collected from each population is given, followed by the number sampled (and successfully used) for DNA content estimation, and cpDNA sequence analysis

	Collection locality	Code	# Samples collected	# Samples in DNA content analysis	# Samples in cpDNA sequence analysis
Commercial	American Commercial	AC	28	21 (10)	16
	Canadian Commercial	CC	37	37 (34)	23
Northern United States	Quonnipaug Lake, Connecticut	CT-A	21	21 (13)	8
	Anderson Pond, Connecticut	CT-B	25	18 (10)	9
	St. Joseph River, Indiana	IN	24	21 (10)	8
	Lake Sabbatia, Massachusetts	MA	31	20 (19)	7
	Barton Lake, Michigan	MI	22	29 (10)	17
	Otternic Pond, New Hampshire	NH	30	27 (19)	15
	Lake Shenandoah, New Jersey	NJ	28	22 (14)	13
	Lower Yaphank Lake, New York	NY-A	27	25 (16)	12
	Jenny Lake, New York	NY-B	25	23 (20)	10
	Cullaby Lake, Oregon	OR	10	6 (2)	0
	Paupackan Lake, Pennsylvania	PA-A	8	8 (4)	5
	Conneaut Lake, Pennsylvania	PA-B	27	27 (15)	7
Southern United States	Ledwith Lake, Florida	FL	8	7 (3)	9
	Cross Lake, Louisiana	LA	20	16 (8)	2
	Pearl River, Mississippi	MS	25	19 (12)	0
	Foster Creek, South Carolina	SC	5	5 (3)	2
	San Marcos River, Texas	TX	5	5 (5)	3
Canada	Kasshabog Lake, Ontario	ON	100	99 (82)	28

### Plastid sequencing

To examine patterns of genetic diversity within and among *C. caroliniana* populations, three noncoding regions of chloroplast DNA (cpDNA) were sequenced in a subset of samples. All three regions (intergenic spacers *atpF*-*atpH*, *trnH*-*psbA,* and *trnL*-*trnF*) are known to exhibit moderate levels of variation in a variety of plant species (Kress et al. 2005; Fazekas et al. 2008; Pleines et al. 2009). Sequences were obtained from 2 to 30 individuals in each population; however, samples from two commercial sources and from OR and MS populations were not included due to poor amplification success. In total, 194 samples from 18 populations (average = 11 per population) were sequenced for all three regions.

Total DNA was extracted from 20 mg of dried leaf tissue per sample using DNeasy Plant Mini Kits (Qiagen, Mississauga, ON, Canada) according to the manufacturer's instructions. Amplification reactions were performed using a 20 μL reaction mixture that contained 5 mmol/L MgCl_2_, GeneAmp PCR Buffer II (Applied Biosystems, Foster City, CA) diluted to 1×, 5% Trehalose, 0.8 U AmpliTaq gold (Applied Biosystems, Foster City, CA), 0.5 mmol/L dNTPs, 2.5 mmol/L MgCl_2_, and 0.1 μmol/L forward and reverse primers and ∼30 ng genomic DNA. The three primer pairs used were previously published (Taberlet et al. 1991; Kress et al. 2005; Fazekas et al. 2008). All reactions were performed on a PTC-200 Thermal Cycler (MJ Research Inc., Waterdown, MA) using a standard polymerase chain reaction (PCR) amplification protocol. Samples were held at 95°C for 3 min, followed by 10 cycles of denaturation at 95°C for 1 min, primer annealing at 58°C for 30 sec, decreasing by 0.5°C every cycle, and extension at 78°C for 1 min. This was followed by 30 cycles of the same protocol at a constant annealing temperature of 53°C. To end, the samples were held at 72°C for 6 min.

Sequencing reactions were conducted in 11 μL volumes containing 5× Sequencing buffer (Tris-HCl and MgCl_2_) and Big Dye v3.1 mix (dNTPs, Tris-HCl, and MgCl_2_; Applied Biosystems), 0.5 mmol/L of either the forward or reverse primer, and 1 μL of the PCR product from the amplification reaction. The sequencing reaction comprised denaturation at 96°C for 2 min, followed by 30 cycles of 30 sec of denaturation at 96°C, primer annealing for 30 sec at 55°C, and extension for 4 min at 60°C. The products of the sequencing reactions were then sent for sequencing on an Applied Biosystems 3730 DNA Analyser at the Genomics Facility at the University of Guelph. Forward and reverse reactions were paired and assembled into sequences using Sequencher v.4.8 (Gene Codes Corporation, Ann Arbor, MI). All bases called by the programs and any inconsistencies between the sequences were manually checked and the primer sequences were removed. All sequences were aligned manually in BioEdit v. 7.0.9.0 (Hall #b^502^). The three regions were concatenated and a statistical parsimony network was created in TCS v1.03 (Clement et al. 2000). For all genetic analyses, base pair indels were coded as single mutation events.

Analysis of Molecular Variance (AMOVA) was performed on the entire haplotype data set to test for genetic differences among populations. We used Arlequin v. 3.1.1 (Excoffier et al. 2005) for this analysis with the default settings and the Kimura-2-Parameter molecular distance. Two additional hierarchical AMOVAs were compared to test the prediction that the Canadian population was genetically more similar to commercial sources than to introduced populations in northern United States. In the hierarchical AMOVAs, populations were grouped into regions, enabling us to estimate Φ_ST_, the variation that exists among populations; Φ_CT_, the variation among the assigned groups of populations; and Φ_SC_, the variation within the assigned groups. The first AMOVA contained three groups of populations: Canadian, introduced United States, and native United States. In this grouping, Canadian and introduced US groups were pooled with their nearest commercial sources (i.e., plants from ON were grouped with CC plants, plants from northern United States were grouped with the AC plants). We compared the magnitude of the molecular variance in this first grouping to a second AMOVA with groups consisting of commercial plants (AC and CC), introduced populations (ON plus northern United States), and native (southern) United States populations. If the Canadian population was derived from a commercial source, we expect the variation in the first grouping to be larger among groups (Φ_CT_) and smaller within groups (Φ_SC_). AMOVA was also performed on the introduced populations only (i.e., populations from the northern United States and Canada), to determine if there was significant genetic structure among them. Pairwise Φ_ST_ values between all populations were also estimated.

### Flow cytometry

As *C. caroliniana* occurs as different ploidy cytotypes (3×, 6×, 8×) (Ørgaard 1991), we tested for variation in DNA content within and between populations using flow cytometry following Doležel et al. (2007). To estimate the DNA content of *C. caroliniana*, nuclear fluorescence was compared to a standard with a known ploidy and genome size, *Pisum sativum* (Doležel and Bartos 2005). Dried plant tissue has been successfully used for flow cytometric estimation of ploidy (Suda and Trávníček 2006) and genome size (Bainard et al. 2011). To confirm if dried *C. caroliniana* tissue could be used, preliminary tests were conducted. Three replicates of each of three plants from each of six commercial stores were analyzed with flow cytometry using both dried and fresh tissue. There were no significant differences in the DNA content estimates obtained from the two treatments (two-tailed *t*-test *t* = 7.52, df = 17, *P* = 0.18).

To isolate the nuclei, four pairs of leaves of each sample of *C. caroliniana* and 2 cm^2^ of leaf of *P. sativum* were cochopped using a razor blade in a modified de Laat's buffer (De Laat and Blaas 1984; Bino et al. 1992; with the addition of 0.25 mmol/L PVP-40). The buffer was chosen after testing several options as it produced data with high nuclei counts and low coefficients of variation (CVs). The homogenate was filtered through a 30 μm mesh and the filtrate was centrifuged for 10 mins at 1200*g*. The supernatant was removed and the pellet was resuspended in the modified de Laat's buffer containing 50 μg/mL of propidium iodide fluorochrome and 50 μg/mL RNAse A for a minimum of 15 mins. Each collected *C. caroliniana* sample was coprepared and analyzed with the *P. sativum* internal standard three times, by analyzing once on three separate days.

Samples were run on a BD Biosciences FACSCalibur flow cytometer (BD Biosciences, San José, CA) for 3 mins using CellQuest Pro software (Becton Dickinson and Co., 1996; http://www.bdbiosciences.com). The FL2 detector (585/42 nm) was used to measure fluorescence, and the parameter FL2-area (integrated fluorescence) was used to quantify DNA content. A typical flow cytometry plot displays fluorescence on the *x*-axis (which is proportional to DNA content) and particle number on the *y*-axis. Additionally, the data were gated on a scattergram of FL2 versus FL3 (670 nm) to remove debris particles that were not part of the standard or the sample peaks. The relative positions of both the *P. sativum* standard and the *C. caroliniana* peaks (Fig. S1) were measured using Modfit (Verity Software House, Inc., 2000; http://www.vsh.com) to remove bias from the procedure. Modfit fits a normal curve to the fluorescence histograms and automatically measures their position and CV. A small proportion (∼10%) of the samples could not be measured in Modfit despite having visible peaks, so these samples were gated manually in CellQuest Pro. The absolute DNA content of the *C. caroliniana* sample was estimated using the known DNA content of 9.09 pg 2C^−1^ for *P. sativum* (Doležel and Bartos 2005) and the formula:


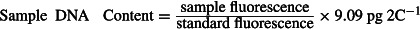
(1)

There was much variation in the quality of the flow cytometry results among samples. To ensure the data met minimum quality standards, we removed all samples with a fluorescence peak CV of more than 6%. While most samples had well over 1000 nuclei in the sample peak, we also set a minimum threshold of at least 300 nuclei. After applying these quality measures, we then eliminated all individuals with <2 replicates. The remaining replicates were averaged, and any individual with a standard error of the mean >0.2 was also removed from the analysis. This threshold allowed us to remove cases where experimental error was deemed to be inappropriately high. Sources of experimental error could be due to degradation of the plant tissue, slight variations in sample preparation or instrument functioning, etc.

We used analysis of variance (ANOVA) to test for differences in mean DNA content among populations and samples nested within populations (random effect). To ensure the data met the assumptions of ANOVA, the residuals were tested for deviations from normality using a Shapiro–Wilk goodness of fit test (*W* = 0.99, *P* < 0.2). The American commercial and Canadian commercial samples were pooled into two populations (AC, CC, respectively) after separate ANOVAs indicated that none of the DNA contents within these groups were significantly different from one another (AC samples: ANOVA, *F*_2, 7_ = 1.19, *P* = 0.36; CC samples: ANOVA, *F*_12, 21_ = 1.88, *P* = 0.09). Tukey–Kramer honestly significant difference (HSD) tests were performed to identify which populations had significantly different mean DNA contents. All analyses were conducted with JMP statistical software (JMP v.8, S.A.S. Institute Inc., 2008).

### Greenhouse experiment

As growth conditions can have minor effects on DNA content estimates (e.g., Price et al. 2000), the population DNA contents for a subset of populations were reassessed after growing plants in a common greenhouse environment. Between two and four plants from each of 14 populations, for which fresh tissue was available (AC, CC, CT-A, CT-B, IN, MA, MI, NH, NJ, NY-A, NY-B, PA-A, PA-B, ON), were grown in the University of Guelph Phytotron for a minimum of 8 months. Plants were submerged in bins of water containing ∼3 inches of gravel (0.5–1 cm in diameter) and First Layer Pure Laterite Aquarium Planting Medium (API Aquarium Pharmaceuticals, Chalfont, PA). Air pumps were used to circulate oxygen, and an aquatic plant fertilizer (Freshwater Plant Formula K–Fe Micronutrient Plant Supplement, Kent Marine, Franklin, WI) was added when plants appeared chlorotic or nutrient stressed. Snails and large algal blooms were manually removed whenever possible. Newly produced tissue from each plant was analyzed for DNA content but replicates were not possible due to small quantities of tissue.

Mean DNA content estimates from paired field and greenhouse samples from each population were compared using ANOVA with population and growth environment as main effects and a population × growth environment interaction term. A Tukey–Kramer HSD test was performed on the least squares means of the population × growth environment data to determine which population means from the two growth environments were significantly different. Because the residuals were nonnormal (Shapiro–Wilk *W* goodness of fit test, *W* = 0.9545, *P* < 0.0001) and the variances of populations were unequal (Levene, *F* = 6.33, df = 13, *P* < 0.0001; Bartlett, *F* = 8.25, df = 13, *P* < 0.0001), nonparametric Welch's ANOVAs were also performed to test whether population and growth environment significantly affected DNA content estimates.

## Results

### cpDNA sequences

**Figure 3:**
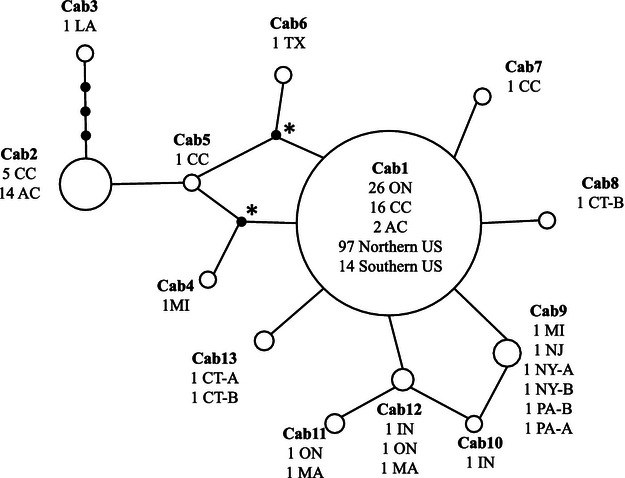
Statistical parsimony network of all three sequenced regions combined (intergenic spacers *atpF-atpH*, *trnH-psbA,* and *trnL-trnF*) displaying the final 13 haplotypes (Cab1-13) and the frequency of detection. Small black dots represent missing haplotypes, and those marked with an asterisk are a 4 bp mutation.

Three cpDNA regions (*atpF*-*atpH*, *trnH*-*psbA*, *trnL*-*trnF*) were analyzed from the *C. caroliniana* samples. Over all populations sampled, 221 sequences were successfully obtained for *atpF-atpH*, which was 535 bp long and included three haplotypes. For *trnH-psbA*, 258 sequences were generated for this 314 bp region and four haplotypes were found. The third region, *trnL-trnF*, generated 199 sequences that were 443 bp long and resulted in 10 haplotypes. The haplotype maps for the three regions can be found in the supporting information (Figs. S2, S3, S4). In each of the three regions, one dominant haplotype was exhibited by the majority of samples, followed by a second haplotype found in most of the remaining samples. In *atpF-atpH* and *trnH-psbA*, the major haplotypes were shared by nearly the same set of individuals. The highest sequence variation was observed in *trnL-trnF*. Combining all three sequenced regions into a composite haplotype resulted in a network containing 13 haplotypes (Fig. 3). There were 19 variable sites, including two 4 bp mutations. Most of the samples (∼80%) shared a common haplotype, Cab1 (Fig. 3); 10% shared the next most common haplotype, Cab2.

**Figure 4:**
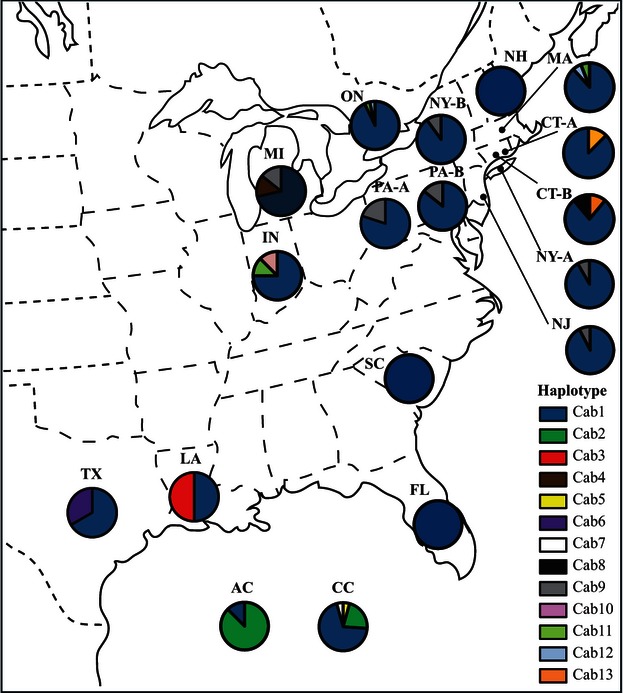
Frequency of plastid haplotypes and DNA contents among populations of *Cabomba caroliniana* from Canada, northern United States, southern United States, and commercial sources. Location codes can be found in Table 1.

There was variation in the haplotype composition of the four geographic groups of populations in the study: commercial, northern United States (introduced), southern United States (native or naturalized), and Canada (Fig. 4). Starting with the commercial samples, most haplotypes were Cab1 and Cab2, though CC samples had a higher proportion of Cab1 (70.0% of samples) and AC samples had more of Cab2 (87.5%). Canadian commercial samples also had two unique haplotypes, Cab5 and Cab7 (Fig. 3). In the northern United States, there was a dominance of the Cab1 haplotype, along with seven other haplotypes (Cab4, 8, 9, 10, 11, 12, 13) (Fig. 4). Several of these haplotypes were limited to the northern United States: Cab9 occurred at low frequency in 6 of the 11 northern populations, Cab4 and Cab10 were unique to Michigan and Indiana, respectively, Cab13 was shared by the two populations in Connecticut and Cab8 was unique to CT-B. The southern United States populations contained three haplotypes (Cab1, Cab3, Cab6). Two of the haplotypes were unique to the south, Cab3 in Louisiana and Cab6 in Texas. Cab3 is unusual in that it is genetically most similar to Cab2, the second-most common haplotype, which contains only commercial plants. In the Canada (ON) population, three different haplotypes were found, including the most common haplotype, Cab1. There were no unique haplotypes in the Canadian population, however, two rare haplotypes (Cab11, Cab12) were present, both of which also occurred at low frequency in the Massachusetts population. This is the only example of two rare alleles being shared between regions and between populations.

The AMOVA of the complete data set revealed significant genetic (sequence) differentiation among populations (Φ_SC_ = 0.41, *P* < 0.001, df = 17,176). Hierarchical AMOVAs indicated that the Canadian population was more similar to populations in the northern United States than to commercial sources. When the Canadian population was grouped with those from the northern United States, the variance among regions, populations within regions, and within populations were all significant (Φ_SC_ = 0.23, *P* < 0.0001; Φ_ST_ = 0.53, *P* < 0.0001; Φ_CT_ = 0.39, *P* < 0.05). When the Canadian population was grouped with the commercial population, the variance among regions was not significant (Φ_SC_ = 0.45, *P* < 0.0001; Φ_ST_ = 0.36, *P* < 0.0001; Φ_CT_ = −0.15, *P* = 0.98). While grouping ON with the northern United States did not explain all the variation between groups (Φ_SC_ was still significant), it better explained the variation than a commercial introduction. When an AMOVA was run exclusively on the populations from the northern United States there were no significant differences between populations (Φ_ST_ = −0.006, *P* = 0.54, df = 11,127). Population pairwise Φ_ST_ values indicated that the Canadian population was significantly different from both American commercial and Canadian commercial populations but not from any wild populations.

### DNA content

**Figure 5:**
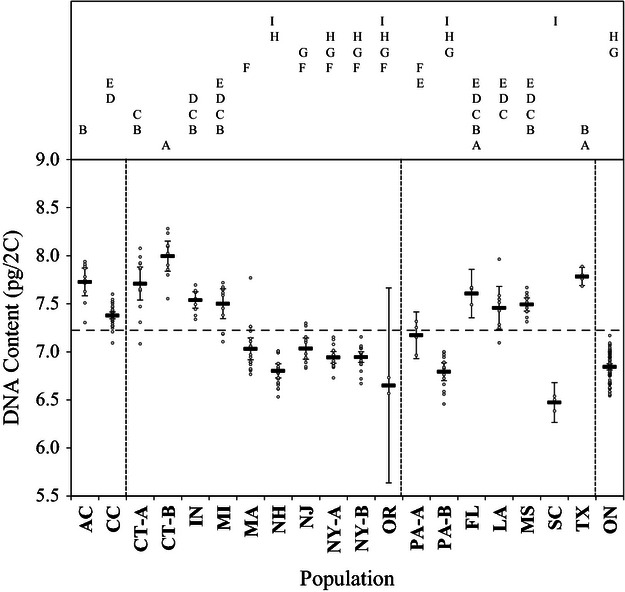
DNA content (pg 2C^−1^) for 20 populations of *Cabomba caroliniana* from North America. Individual estimates are represented by gray circles and the black bar represents the population mean ± 95% confidence limit (error bars). The horizontal dashed line represents the threshold between low and high DNA contents. Vertical lines separate the population groups (commercial, northeastern U.S., southern U.S., and Canada). Populations represented by the same letter above the figure are not significantly different from each other (Tukey–Kramer HSD).

Individual DNA content estimates ranged from 6.38 pg 2C^−1^ to 8.28 pg 2C^−1^, while population means ranged from 6.47 pg 2C^−1^ to 8.00 pg 2C^−1^ (Fig. 5). There were significant differences in mean DNA content among populations (*F*_19,286_ = 79.04, *P* < 0.0001; Welch's ANOVA for heterogeneous variances *F*_19,106_ = 113.28, *P* < 0.0001). Based on Tukey–Kramer HSD means comparisons (Fig. 5), the populations fell into two DNA content classes. These classes will be henceforth referred to as “high” DNA content populations (mean between 7.4 pg 2C^−1^ and 8.0 pg 2C^−1^) and “low” DNA content populations (mean between 6.5 pg 2C^−1^ and 7.2 pg 2C^−1^). All the populations with low DNA content except PA-A were significantly different from the populations with high DNA content (Fig. 5). As the two categories are not absolute and we don't yet understand the genetic basis for these differences, we use them here for comparison to population similarities based on cpDNA.

DNA content was relatively homogeneous within populations. There were no differences in DNA content among samples within 14 populations. Two populations each contained one individual that differed from one other, which may arise through measurement error. Four populations (MA, LA, CT-A, CT-B) had more heterogeneity among individuals, and three of these populations contained a minority of estimates from the alternate DNA content category. There was no difference among samples within the Kasshabog Lake (ON) population (ANOVA, *F*_81, 143_ = 1.15, *P* = 0.23).

**Figure 6:**
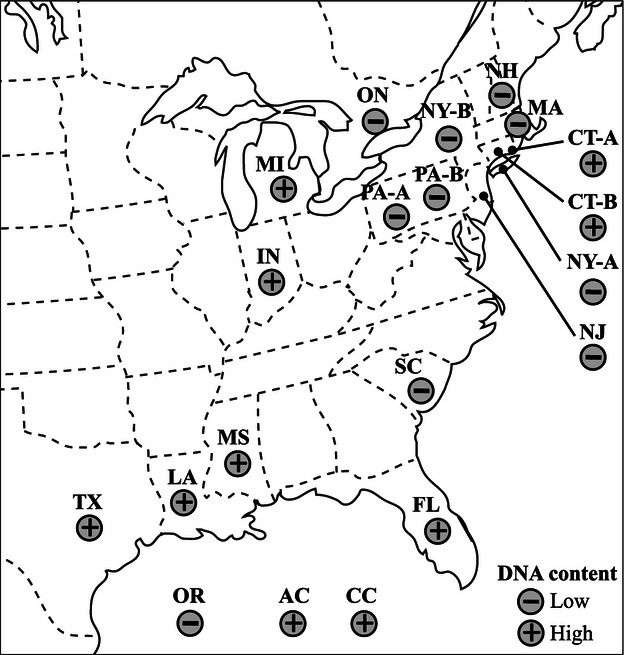
Geographic distribution of DNA content for *Cabomba caroliniana* in North America. “Low” DNA content = 6.5–7.2 pg 2C^−1^; “High” DNA content = 7.4–8.0 pg 2C^−1^. Location codes can be found in Table 1.

There was geographic variation in DNA content (Figs. 5, 6). The commercial populations (AC and CC) had relatively high DNA contents (7.4 pg 2C^−1^ and 7.7 pg 2C^−1^, respectively) and fell within the high DNA content category. Populations in the northern United States had both high (four populations) and low DNA contents (eight populations). The high DNA content populations in this region were split geographically, with two in the northeast (CT-A, CT-B) and two in the northwest (MI, IN). Of the five populations in the southern region, all had high DNA content except for SC (Fig. 6). With a mean DNA content of 6.84 pg 2C^−1^, the Canadian population (ON) belonged to the low DNA content category. ON was not different from the eight low DNA content populations in the northern U.S. or from SC in the south, but was significantly different from all populations with high DNA content, including both commercial populations (Fig. 5). The single west coast population (OR) had a low DNA content although the small sample size contributed to a large variance.

The greenhouse experiment revealed significant variation in DNA content between plants grown in both greenhouse and natural environments. ANOVA indicated a significant effect of population (*F* = 50.08, sum of squares = 29.46, df = 13, *P* < 0.0001), growth environment (*F* = 69.16, sum of squares = 3.13, df = 1, *P* < 0.0001), and population × growth environment interaction (*F* = 1.74, sum of squares = 2.96, df = 13, *P* < 0.001). On average, greenhouse grown plants (mean = 6.92 pg 2C^−1^) had a lower DNA content than naturally grown plants (mean = 7.16 pg 2C^−1^). However, Tukey HSD comparisons for each population indicated that only two populations (MA and NJ) had means that were significantly different between growth environments. More important for this study, the population designation of high and low DNA content did not change for any population based on plants grown in a common environment for 8 months.

## Discussion

The chloroplast sequence data and the results of the hierarchical AMOVA reveal that *C. caroliniana* plants from ON are more similar to other individuals from the invasive range in the northern United States than they are to plants obtained from commercial sources. As the *C. caroliniana* samples were dominated by a single common haplotype, rare haplotypes were used to infer source populations (Slatkin 1985). The Canadian population (ON) contained plants with two rare haplotypes (Cab 11, Cab12) that were shared with plants from the Massachusetts (MA) population. Although Cab12 was also present in Indiana (IN), no other populations sampled in the United States contained Cab11, and MA and ON are the only two populations containing them in combination. In a species that is expanding geographically rapidly and recently, two populations that share rare alleles more likely reflect pathways of geographic expansion as opposed to these alleles arising independently (Slatkin 1985). However, given the presence of common haplotypes in Kasshabog Lake, we are unable to eliminate the possibility of multiple introductions from other sources though it seems less likely.

DNA content was highly variable among individuals and populations of *C. caroliniana*. The absence of information about its underlying cause makes it difficult to conclusively infer historical relationships among populations. With that said, there were strong differences between mean DNA content of high and low groups (Fig. 5), which were supported statistically and can be compared to patterns based on cpDNA. Importantly, the DNA contents of ON plants were similar to several populations in the northern United States, including MA, but significantly different from the commercial plants.

The two major classes of DNA content (high and low) differed by roughly 11%, which could be due to variation in chromosome number. If the observed difference in DNA content was due to a difference in ploidy (i.e., number of copies of complete chromosome sets), the difference is expected to be roughly double (100%) in the case of a triploid compared to a hexaploid or octaploid, and ∼33% when comparing a hexaploid to an octaploid, yet this is not the case. However, genome downsizing is known to occur in many polyploid species, and plants with higher ploidy levels don't always have DNA contents at the expected increments (or may even have a lower 2C-value than their diploid relatives; Leitch and Bennett #b^501^). Another potential source for the variation in DNA content is aneuploidy, which is not unexpected in this group given the previously reported range in chromosome number (Ørgaard 1991). New chromosome counts from the populations studied are imperative to fully understand the variation in DNA content in North American *C. caroliniana*. Hybridization has not been documented within *Cabomba* (Wilson et al. 2007) but cannot be ruled out as a source of variation in DNA content. Hybridization between parents with differing DNA contents can result in progeny with genome sizes that are either larger (e.g., Baack et al. 2005) or intermediate (e.g., Marques et al. 2012) as compared to the parents.

Evidence for intraspecific variation in genome size among plant and animal species is sparse and is often attributed to methodological artifacts (Greilhuber 2005). For example, growth environment is known to cause intraspecific variation in DNA content by affecting the concentration of secondary compounds in leaves, which in turn alters propidium iodide stain uptake (e.g., Price et al. 2000; Walker et al. 2006). In this study, there were significant differences in DNA content between plants grown in a common environment and those collected from natural environments, which may account for some of the variation seen between and within populations. However, the high and low DNA content categories in *C. caroliniana* remained unchanged after plants were grown in a common environment, and two similar classes of DNA content were also observed in a preliminary study of *C. caroliniana* (M. C. Miller and B. C. Husband, unpubl. data). Intraspecific genome size differences (not due to methodology or ploidy) have been reported previously (Šmarda 2006; Šmarda and Bureš 2006; Loureiro et al. 2007). There are several examples of genome size variation due to reproductive isolation (Eilam et al. 2008) and population isolation (Achigan-Dako et al. 2008; Pellicer et al. 2009; Slovak et al. 2009). In these examples, differences in genome size may be the result of independently evolving lineages, which may be the case in *C. caroliniana*.

**Figure 7:**
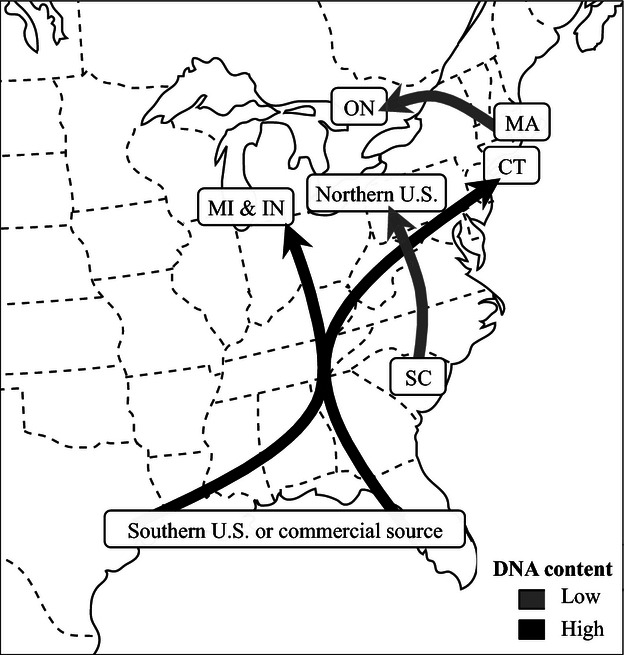
Schematic diagram of possible pathways of introduction of *Cabomba caroliniana* in North America.

The geographic distribution of cpDNA and DNA content in North American *C. caroliniana* populations suggests there have been a minimum of three introductions into the northeastern United States (Fig. 7). One hypothesized introduction of *C. caroliniana* is represented by the two populations from Connecticut, and a second introduction is represented by the Michigan and Indiana populations. These four populations in the northern United States (CT-A, CT-B, IN, and MI) are possibly derived from populations with high DNA content in the southern United States or from a commercial source. In fact, there are reports of *Cabomba* being intentionally introduced in Michigan in the 1890's (Les and Mehrhoff 1999). A third introduction in the northeastern United States (of the low DNA content lineage) may have arisen from a natural population in the native range in the southern United States, such as the population in South Carolina (Fig. 7). Alternatively, as early reports of introduced *C. caroliniana* date back to over 70 years ago, there could have been a different commercial source at that time (Les and Mehrhoff 1999; Mehrhoff et al. 2003). There is a slight chance that a pathway exists from IN to the other northeastern populations (based on sharing Cab12) but this scenario seems unlikely given the differences in DNA content and the absence of the other rare haplotype. The pattern of multiple introductions and subsequent spread of populations of *C. caroliniana* in the United States is also supported by several observational and herbarium records (e.g., Les and Mehrhoff 1999; Mehrhoff et al. 2003).

Some conclusions about commercial sources of *C. caroliniana* and pathways of introduction can also be drawn from the cpDNA sequences. The haplotype networks indicate that most commercial plants have one of two genetically divergent haplotypes. While the majority of CC plants have the most common haplotype (Cab1) found in natural populations, 87.5% of the AC samples contain a haplotype (Cab2) not present in natural populations. This makes American commercial trade an unlikely source of *C. caroliniana* in natural populations, assuming the genetic composition of commercial plants has remained unchanged from when the plants were originally introduced. In addition, the difference in haplotype composition between United States and Canadian commercial outlets indicates that there are multiple suppliers of commercial plants and that the distribution of plant material to retail outlets is regionally based. Commercial operations are often implicated as sources of invasive species in cases where the species is commercially available (e.g., Wilson et al. 2007; Ugelvig et al. 2008). Our results suggest that allegations such as these should be tested empirically, especially when there are plausible alternatives. Assumptions of commercial release have been proven to be incorrect in other invasive species (e.g., Booth et al. 2007; Cunningham 2008).

Excluding a commercial release, the most likely source of *C. caroliniana* in Canada is due to accidental transfer, perhaps via boat traffic. Boat traffic between invaded and noninvaded lakes has been previously cited as a vector for invasive species (e.g., Muirhead and MacIsaac 2005). As Kasshabog Lake is surrounded by cottages and is frequently used by recreational boaters, this is a likely possibility (Les and Mehrhoff 1999; Wilson et al. 2007). Natural introductions cannot be discounted, as migrating waterfowl have been known to transport multiple aquatic plant species (Cook 1990; Madeira et al. 2000). However, waterfowl are often reported as moving seeds, and in the northern United States *C. caroliniana* appears to have low seed set and low seed viability (Wilson et al. 2007), making human-mediated dispersal more likely.

Multiple introductions appear to be common in invasive species and are believed to have occurred in several other aquatic invasive species (e.g., Provan et al. 2005; Booth et al. 2007; Lejeusne et al. 2011; Thum et al. 2011). A similar genetic pattern to *C. caroliniana* was found in an analysis of mitochondrial haplotypes in an invading ascidian *Microcosmus squamiger* in Australia. Although all populations contained two common haplotypes, indicating one or two common sources, there was support for subsequent smaller colonizations indicated by populations sharing rare alleles (Rius et al. 2008). This pattern of a small number of long-distance dispersal events followed by localized regional dispersal has been observed in several other invasive species (e.g., Bartlett et al. 2002; Genton et al. 2005; Ugelvig et al. 2008), and indicates that geographic scale may be important when monitoring source populations.

Our results have important implications for the management of *C. caroliniana* in Canada and potentially elsewhere. Some of the original introductions of *C. caroliniana* in the United States may have been from commercial sources, and this pathway should not be excluded from consideration in any potential management plan. However, much of the subsequent spread of *C. caroliniana* is not from commercial sources, and banning the sale of this plant will likely be ineffective at stopping its spread. A risk assessment of Ontario lakes indicates that *C. caroliniana* is at high risk to spread throughout the region based on both habitat suitability and estimates of boater traffic (Jacobs and MacIsaac 2009). Warnings to boaters about the importance of cleaning their boats to stop the spread of invasive aquatics, along with the inspection of boats leaving and entering lakes or border crossings may be essential in controlling the spread of *C. caroliniana*.

In the future, genetic analyses such as this one should be conducted elsewhere in *C. caroliniana's* introduced range and should include extensive sampling of the native range and a wider sampling of commercial sources. This might provide some information about when and where commercial releases are occurring. This type of study should also be conducted for other introduced species, especially those presumed to have commercial sources. If invasive populations more commonly arise from previously introduced populations as opposed to novel introductions from a commercial source, then there are significant implications for how these species are managed and controlled. More research is needed to test the fundamental assumptions made about the spread of invasive species.

## Data Accessibility

DNA sequences for combined haplotype network: Genbank accessions KC525061 – KC525077.

## Supporting Information

**Figure S1.** Example flow cytometry histogram for determining 2C DNA content of *Cabomba caroliniana*.

**Figure S2.** Statistical parsimony haplotype network of *atpFatpH*. The number of representatives from each geographic region is provided. Black dots represent missing haplotypes.

**Figure S3.** Statistical parsimony haplotype network of *trnHpsbA*. The number of representatives from each geographic region is provided. Black dots represent missing haplotypes.

**Figure S4.** Statistical parsimony haplotype network of *trnLtrnF*. The number of representatives from each geographic region is provided. Black dots represent missing haplotypes.
