# Supplementary material for: Pathways of introduction of the invasive aquatic plant Cabomba caroliniana
Source: Ecol Evol. 2013 Apr 15;3(6):1427–39. doi: 10.1002/ece3.530 (PMC3686180; doi:10.1002/ece3.530)
Supplement: Supplementary file 1 [file ece30003-1427-SD1.pdf]

Pathways of introduction of the invasive aquatic plant *Cabomba caroliniana*

A. McCracken, J.D. Bainard, M.C. Miller and B.C. Husband

Supporting Information

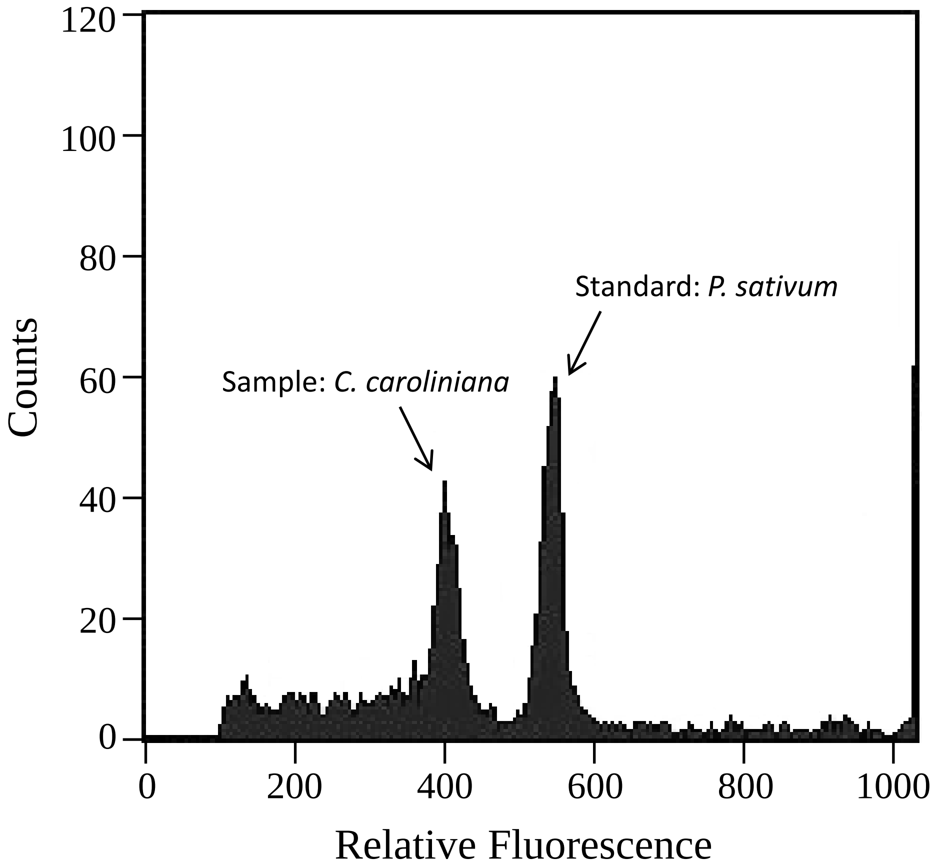

Figure S1. Example flow cytometry histogram for determining 2C DNA content of *Cabomba caroliniana*.

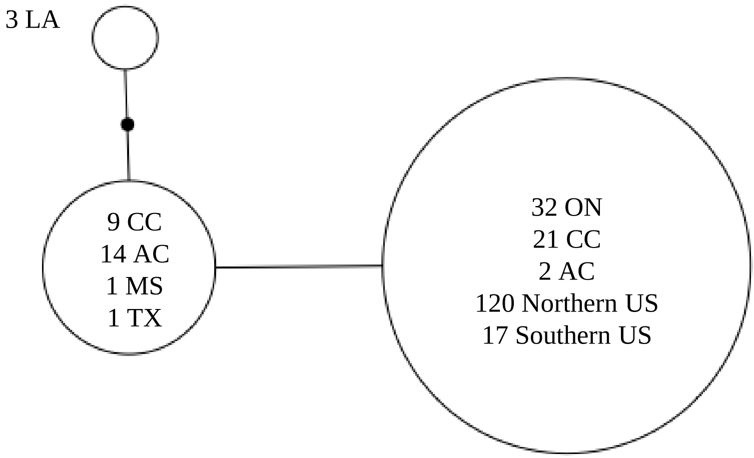

Figure S2. Statistical parsimony haplotype network of *atpF-atpH*. The number of representatives from each geogrphic region is provided. Black dots represent missing haplotypes.

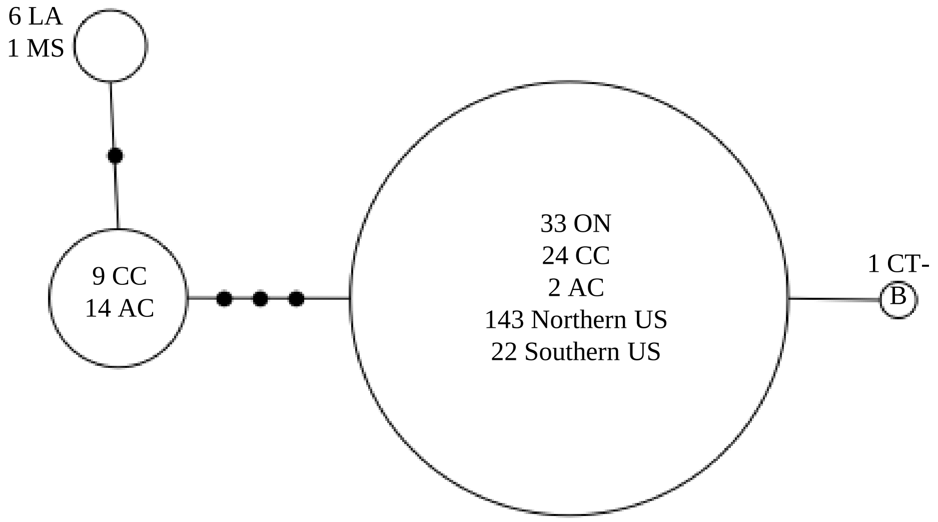

Figure S3. Statistical parsimony haplotype network of *trnH-psbA*. The number of representatives from each geogrphic region is provided. Black dots represent missing haplotypes.

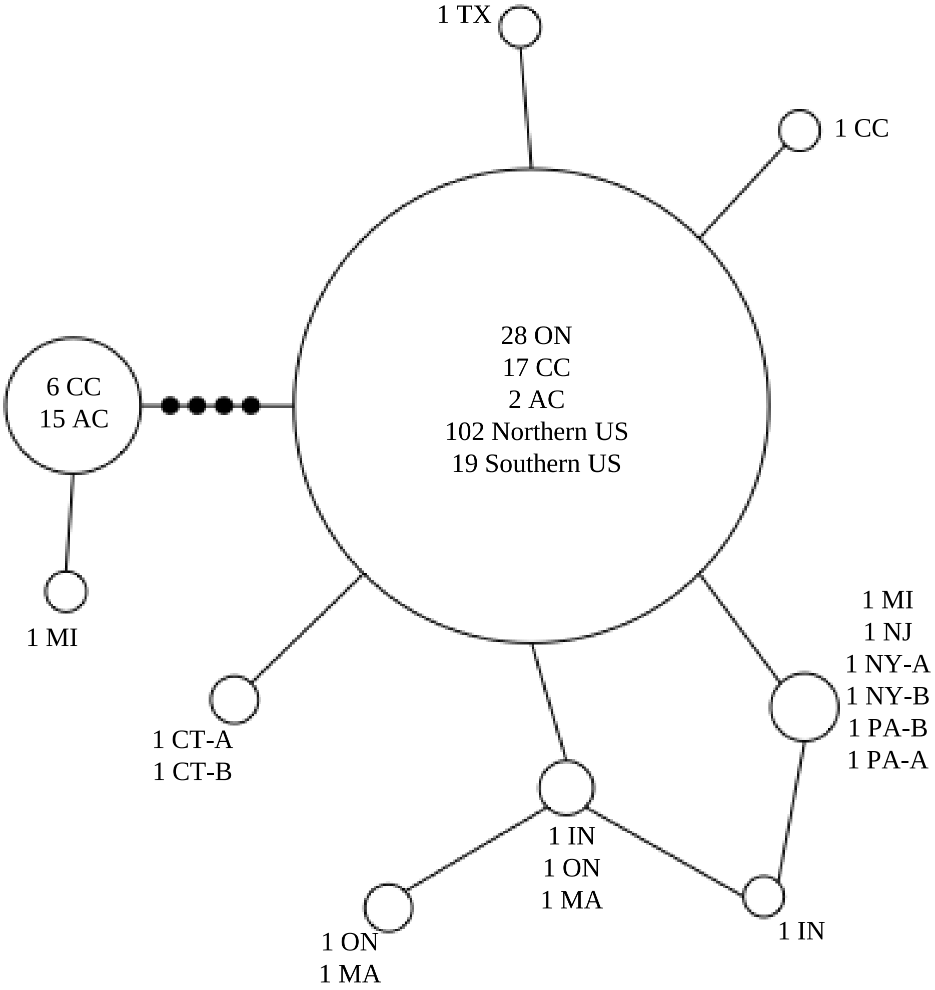

Figure S4. Statistical parsimony haplotype network of *trnL-trnF.* The number of representatives from each geogrphic region is provided. Black dots represent missing haplotypes.
